# Assessing temperature dependence of T1 in cortical bone using ultrashort echo-time MRI

**DOI:** 10.1186/2050-5736-3-S1-P5

**Published:** 2015-06-30

**Authors:** Misung Han, Serena Scott, Eugene Ozhinsky, Vasant Salgaonkar, Peter Jones, Peder Larson, Chris Diederich, Roland Krug, Viola Rieke

**Affiliations:** 1 University of California at San Francisco, San Francisco, California, United States

## Background/introduction

MR-guided high-intensity focused ultrasound (HIFU) ablation is a promising, noninvasive method for treatment of bone tumors and palliation of pain.

During thermal therapy, temperature mapping is necessary to ensure proper heat deposition in targeted tumors as well as to prevent undesired heating in healthy tissues. However, conventional proton resonance frequency-based MR thermometry cannot be applied in cortical bone due to its short T2* relaxation time. Recently, it was shown that ultrashort echo-time MRI can be used to assess T1 change in cortical bone due to temperature change (Han M, ISMRM 2014, P262). This work focused on characterization of temperature dependence of T1 in cortical bone by performing a calibration experiment.

## Methods

A calibration experiment was conducted using cortical bone samples from diaphysis segments of bovine femurs. Two bone samples were placed in a container filled with circulating water controlled by a water bath. To monitor temperature, fiber optic temperature sensors (Luxtron, LumaSense Technologies, Santa Clara, CA) were placed, with one sensor inside the water bath and one in a bone sample. Imaging was performed at a GE discovery MR750 widebore 3T scanner (GE healthcare, Milwakee, WI) using a 16-channel large flex coil (GE healthcare, Cleveland, OH). Cortical bone T1 was measured at temperatures of approximately 25, 35, 45, 55, 65, and 70°C during the heating phase and 55, 35, and 25°C during the cooling phase after the cortical bone samples and the water reached thermal equilibrium. For T1 mapping, 3D UTE imaging was performed by incorporating a nonselective hard pulse with a 200 μs duration followed by 3D radial imaging. The scan parameters included a 75 μs TE, 11 ms TR, and 1.7 mm isotropic resolution. To measure T1 using a variable flip angle scheme, UTE imaging was performed at 8° and 44° flip angles, resulting in a total 8 min scan time.

## Results and conclusions

**Figure 1:**
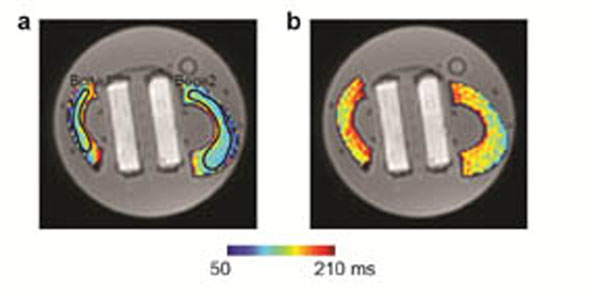
(a-b) Bone T1 maps at temperatures of 25.1°C and 70.1°C, respectively, overlaid on a UTE image. Regions of interest used for analysis are depicted.

**Figure 2:**
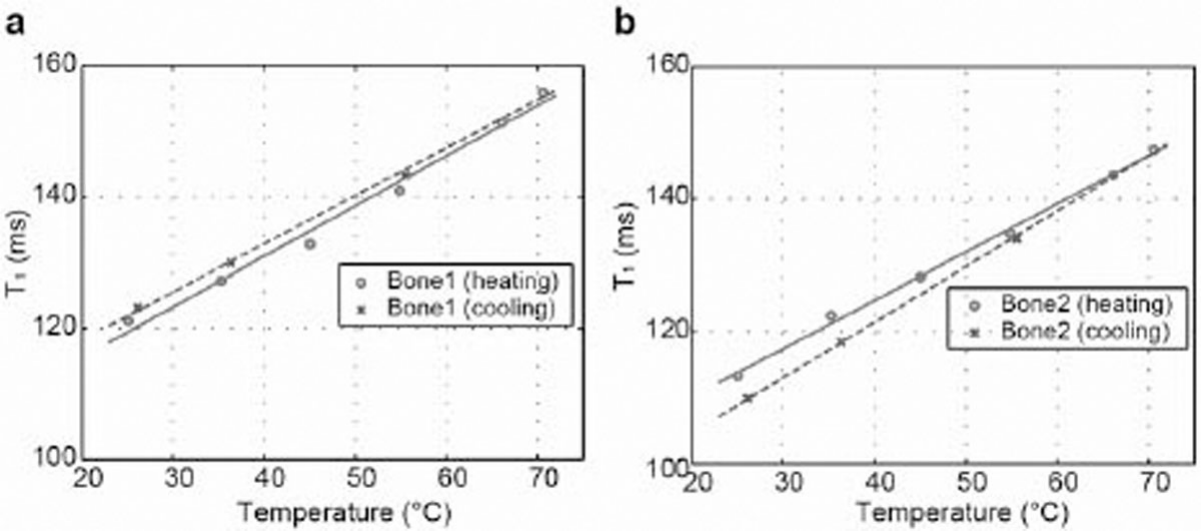
(a-b) T1-temperature plots for two bone samples, respectively. Linear regression lines during heating and cooling are denoted as well.

Our calibration results are shown in Figs 1-2. Figure 1a-1b shows T1 maps at two different temperatures, 25.1°C and 70.1°C, overlaid on a UTE image. Figure 2a-2b shows average T1 values from regions of interest on twelve slices over temperature values measured from the fiber optic sensor. A linear relationship between temperature and T1 was observed for both bone samples. For the first bone sample, the linear coefficient was 0.77 ms/°C during heating and 0.73 ms/°C during cooling; for the second bone sample, it was 0.73 ms/°C during heating and 0.84 ms/°C during cooling. Although the linear coefficients were not identical for heating and cooling, the T1-temperature plots from the two bone samples indicated reversibility in T1 variation. The variable flip angle method is very sensitive to B1 inhomogeneities and flip angle calibration errors, and thus can result in significant T1 measurement errors. Still, our experiment demonstrated a linear temperature dependence of T1 in cortical bone for a temperature range of 25°C and 70°C. By improving accuracy in T1 measurement, direct quantification of temperature changes in cortical bone could be possible, and thermal dose might be more accurately monitored during thermal ablation of bone tumors.

